# Bacterial Production of Indole Related Compounds Reveals Their Role in Association Between Duckweeds and Endophytes

**DOI:** 10.3389/fchem.2018.00265

**Published:** 2018-07-12

**Authors:** Sarah Gilbert, Jenny Xu, Kenneth Acosta, Alexander Poulev, Sarah Lebeis, Eric Lam

**Affiliations:** ^1^Department of Plant Biology, Rutgers University, New Brunswick, NJ, United States; ^2^Department of Microbiology, University of Tennessee, Knoxville, TN, United States

**Keywords:** auxin, *Wolffia*, duckweeds, indoles, Salkowski assay, endophytes

## Abstract

Duckweed farming can be a sustainable practice for biofuel production, animal feed supplement, and wastewater treatment, although large scale production remains a challenge. Plant growth promoting bacteria (PGPB) have been shown to improve plant health by producing phytohormones such as auxin. While some of the mechanisms for plant growth promotion have been characterized in soil epiphytes, more work is necessary to understand how plants may select for bacterial endophytes that have the ability to provide an exogenous source of phytohormones such as auxin. We have isolated and characterized forty-seven potentially endophytic bacteria from surface-sterilized duckweed tissues and screened these bacterial strains for production of indole related compounds using the Salkowski colorimetric assay. Indole-3-acetic acid (IAA), indole-3-lactic acid (ILA), and indole produced by various bacterial isolates were verified by mass spectrometry. Using the Salkowski reagent, we found that 79% of the isolated bacterial strains from our collection may be capable of producing indole related compounds to various extents during *in vitro* growth. Of these bacteria that are producing indole related compounds, 19% are additionally producing indole. There is an apparent correlation between the type of indole related compound produced by a particular bacteria and the duckweed genus from which the bacterial strain is derived. These results suggest the possible association between different duckweed genera and endophytes that are producing distinct types of secondary metabolites. Understanding the role of indole related compounds during interaction between endophytes and the plant host may be useful to help design synthetic bacterial communities that could target specific or multiple species of duckweed in the future to sustainably enhance plant growth.

## Introduction

Duckweeds are aquatic plant species that preferentially reproduce via asexual propagation and are found all over the world. Their fast growth rate and ability to remove excess nitrogen and phosphate pollutants makes them attractive for use in wastewater treatment (Korner and Vermaat, 1998). In addition, duckweed biomass can be harvested for biofuel production or as animal feed additives, since strains with high levels of starch or protein can be identified (Cheng and Stomp, 2009). With the increasingly urgent need for low cost wastewater treatment, alternative energy sources, and increased food supply to support the growing world population, duckweed is a promising area of research that can have an impact on these challenges. However, large-scale production of duckweed continues to be a limiting factor for growing duckweed at wastewater treatment facilities and other production scenarios. In nature, duckweed grows rapidly in stagnant ponds during the summer months. However, man-made duckweed ponds often are susceptible to algae growth and must be harvested frequently to maintain optimal biomass production.

Studying duckweed associated bacteria (DABs) may provide an important management tool for large-scale production of duckweed biomass reliably and sustainably. In general, plant growth promoting bacteria (PGPB) have been shown to improve plant health by increasing nutrient availability, providing defense against pathogens, protection from abiotic stresses, and producing phytohormones such as auxin (Santoyo et al., 2016). Recent studies have revealed that DABs can improve the growth of duckweed by increasing frond production rate as well as higher chlorophyll content (Yamaga et al., 2010; Suzuki et al., 2014). Some duckweed associated bacterial strains were found to breakdown phenol contaminants or increase the removal of nitrogen and phosphate from wastewater by these aquatic plants, which may be correlated with their ability to promote plant growth (Yamaga et al., 2010; Suzuki et al., 2014). After inoculation of a PGPB isolated from the surface of the duckweed, *Lemna aoukikusa* (indistinguishable from *Lemna aequinoctialis* using atpF-atpH and psbK-psbI barcodes Borisjuk et al., 2015), there was an observed increase in chlorophyll content in the monocotyledon *Lemna minor* as well as the dicotyledon *Lactuca sativa* (Suzuki et al., 2014). Thus understanding which microbes promote plant growth and the mechanisms they use to do so, could help establish a more sustainable approach of increasing duckweed biomass as well as the growth of other plants.

There is evidence that the plant phytohormone, auxin, may act as a signal molecule between bacteria and plants (Bianco et al., 2006; Lui and Nester, 2006; Spaepen et al., 2007). IAA is the most common auxin found in nature. It is suggested that over 80% of rhizosphere bacteria may be capable of synthesizing IAA (Spaepen and Vanderleyden, 2011). In certain cases, high concentrations of IAA production by bacteria may increase overall root biomass, allowing the plant to better uptake water and minerals, which in turn can enhance bacteria colonization (Spaepen and Vanderleyden, 2011). Indole is a less common, naturally occurring compound that has been shown to be a signal molecule between microbes in biofilm formation and quorum sensing (Martino et al., 2003; Lee and Lee, 2010). In contrast to the case of IAA, the role of various indole-related compounds during plant-microbe interactions is poorly understood. Therefore, our aim in this work was to focus on indole related compounds as an initial screen to identify plant growth promoting duckweed-associated bacteria (DABs). Future work may involve screening for effects on the production of other plant hormones such as gibberellin or ethylene by strains in our collection. In this work, we have characterized a set of 47 duckweed-associated bacteria (DABs) based on their production of indole related compounds by using the Salkowski reagent and mass spectrometry. The Salkowski reagent consists of ferric chloride and perchloric acid that when mixed with IAA produces a pink color (Gordon and Weber, 1951). Well characterized *Azospirillum brasilense* strains Sp7 and Sp245, originally isolated as plant-associated bacteria from wheat tissues (Bashan and de-Bashan, 2010), were used in our work as positive controls since they are known to produce IAA and one is an epiphyte (Sp7) while the other is an endophyte (Sp245) in wheat. Analysis of duckweed associated endophytes reveals that the production of indole is correlated with the DAB being associated with the *Wolffia* genus whereas the production of indole related compounds such as IAA is correlated with those DABs that were isolated from the *Lemna* genus. Indole and indole related compounds, IAA and indole-3-lactic acid (ILA), were detected from cultures of a subset of DABs in our collection and their potential role in improving duckweed plant health is discussed.

## Materials and methods

### Duckweed tissue

Plant material was obtained from the Rutgers Duckweed Stock Cooperative at Rutgers University, New Brunswick, NJ, USA. Plant strains were maintained in 60 × 15 mm Petri dish on 0.5X Schenk and Hildebrandt (SH) Basal Salt Mixture supplemented with 0.8% w/v Difco Agar, granulated. Plants were additionally maintained on 0.5X SH medium with 0.8% w/v agar and 0.5% sucrose with and without the addition of 100 mg/L cefotaxime. The plants were grown at a temperature of 15°C under illumination of 40–44 μmol m^−2^ s^−1^ light. Duckweed strains stored at the Rutgers Duckweed Stock Cooperative originate from various locations in the world (Supplementary Table 1). Duckweed strains were also collected June 2015 from various locations in New Jersey, USA (Supplementary Table 1).

### Isolation of bacteria

To isolate duckweed associated bacterial strains, duckweed tissue was surface sterilized before placing on LB (Miller) agar medium or Tryptic Soy Agar (TSA). Duckweed tissues were rinsed in 1.5 mL eppendorf tubes with sterile Rinse Solution (0.1% Triton-X, 137 mM NaCl, 2.7 mM KCl, 10 mM Na_2_HPO_4_·7H_2_0, 1.8 mM KH_2_PO_4_, 0.5 mM MgSO_4_, 1 mM CaCl_2_, pH 7.4) for 1 min and then decanted. For isolation using bleach, an extra step was performed by washing the tissue in 0.6% (v/v) sodium hypochlorite until only the meristem retained chlorophyll and then washing with 2% Na_2_S_2_O_3_ for 1 min to neutralize the bleach. The solution was decanted and the tissue was washed in sterile Millipore water for 1 min. The tissue was then homogenized using a plastic drill and then spread onto LB agar plates or TSA plates. The plates were incubated at 28°C for up to 72 h. Colonies on the plates were picked and re-streaked several times on LB or TSA plates for single colonies to obtain pure isolates. Bacterial strains were stored at −80°C in LB or Tryptic Soy Broth (TSB) supplemented with 40% sterilized glycerol.

### Fluorescent microscopy

To confirm that the bleach treatment that we employed to isolate candidate endophytic bacteria from duckweed can effectively remove most if not all surface-associated bacteria, fluorescent microcopy was performed on *Lemna minor* strain 370-DWC112 inoculated with DAB 1A (Supplementary Figure 1). Plant tissue was treated with 0.3% (v/v) sodium hypochlorite for 2 min using the method described for isolation of bacteria, which left only the meristem to retain chlorophyll. The tissue was placed in a microcentrifuge tube with 100 μL of 6 μM Syto 9 stain (ThermoFisher Scientific, Waltham, MA). The Syto 9 stain was removed after 3 min and the tissue was washed twice with 200 μL of sterile water. The tissue was then placed on a microscope slide and observed using an Olympus FSX100 epifluorescence microscope (460–495 nm excitation/510–550 nm emission). To image the surface of the plant tissue, the 10x objective lens was first used to focus on the guard cells of the stomates before switching to higher magnification objectives to resolve stained bacteria on the same focal plane.

### Genotyping duckweed strains

Plant DNA was isolated by grinding 100 mg fresh weight of the tissue in liquid nitrogen and 500 μL of 1x CTAB buffer with 5 μL of 2-mercaptoethanol. The extract was incubated at 65°C for 30 min. 1x volume of phenol/chloroform/isoamyl alcohol (25:24:1) was added. The extract was centrifuged at 10,000 × g for 5 min and then the aqueous phase was transferred into a new tube. Two microliter of RNase A (10 mg/mL) was added and the sample was incubated at room temperature for 30 min. The phenol/chloroform/isoamyl alcohol step was repeated one more time before adding 0.5x volume of 7.5 M ammonium acetate and 2.5x volume of 100% ethanol to the aqueous phase. The sample was centrifuged at 16,000 × g for 30 min at room temperature. The supernatant was decanted and 500 μL of 70% ethanol was added. The sample was centrifuged at 10,000 × g for 5 min and the supernatant was decanted. The pellet was air dried and then resuspended in 5 μL of sterile Millipore water. Concentration and quality of DNA was determined with a Nanodrop-1000 UV/Vis Spectrophotometer (Thermo Scientific, Waltham, MA) and then by running an aliquot of the sample on a 1% agarose gel with ethidium bromide followed by visualization on a transilluminator, respectively.

PCR amplification of the *atpH* and *psbK* regions of the plastid genome was performed to identify duckweed collected from the environment (Borisjuk et al., 2015). For the *atpH* gene the forward primer was ACTCGCACACACTCCCTTTCC and the reverse primer was GCTTTTATGGAAGCTTTAACAAT. For the *psbK* locus, the forward primer was TTAGCATTTGTTTGGCAAG and the reverse primer was AAAGTTTGAGAGTAAGCAT. One hundred nanogram of DNA was added to the amplification mixture containing 1x buffer, 0.25 mM dNTPs, 0.4 μM forward primer, 0.4 μM reverse primer, 4 mM MgCl_2_, and 2 U of Taq polymerase. The PCR condition was Stage 1 at 95°C for 5 min, Stage 2 with 35 cycles at 95°C for 30 s, 51°C for 30 s, 72°C for 1 min, and Stage 3 at 72°C for 5 min.

### Sequencing of 16S rRNA gene

PCR amplification of the 16S rRNA gene was performed on single bacterial colonies using the bacterial universal primers e9f forward (GAGTTTGATCCTGGCTCAG) and e926r reverse (CCGTCAATTCCTTTGAGTTT) (Baker et al., 2003; Chakravorty et al., 2007). DNA was added to the amplification mixture containing 1x buffer, 0.2 mM dNTPs, 0.4 μM forward primer, 0.4 μM reverse primer and 2 U of Taq polymerase. The PCR condition was Stage 1 at 95°C for 5 min, Stage 2 with 25 cycles at 95°C for 1 min, 50°C for 30 s, 72°C for 1 min, and Stage 3 at 72°C for 5 min.

### Sequence analysis

Sanger sequencing of the *atpH, psbK*, and 16S rRNA genes was performed by Genewiz Co. (Piscataway, New Jersey) and sequences were analyzed using Serial Cloner and UGENE programs. Supplementary Table 1 lists the accession numbers for the 16S rRNA sequences registered in Genbank. With the support by the Joint Genome Institute (JGI)-Environmental Molecular Sciences Laboratory (EMSL) Collaborative Science program, the complete genome sequences for 33 of our DAB collection have been determined. Analysis of complete genome sequences was performed using JGI, KEGG Mapper, and RAST annotation service (Aziz et al., 2008; Overbeek et al., 2013; Brettin et al., 2015). Amino acid sequence alignment of tryptophanase and % identity matrix was performed using Clustal Omega version 1.2.4 with default parameters.

### Colorimetric detection of indole related compounds

Bacterial strains from glycerol stocks were streaked onto either an LB agar plate or a TSA plate, depending on the medium of their original isolation and grown at 28°C. For each strain, a single colony was used to inoculate 6 mL of liquid LB medium, with and without 5 mM L-tryptophan. For DAB 33B and DAB 39B, liquid TSB (with and without 5 mM L-tryptophan) was used instead due to difficulty growing these two strains on LB medium. After 48 h of growing at 28°C with shaking at 240 rpm, 1 mL of culture was centrifuged for 5 min at 14,000 rpm to collect the supernatant. The original Salkowski assay based on the Gordon and Weber protocol was adapted for a 96-well format (Gordon and Weber, 1951). In a Corning 96-well clear bottom white plate, 100 μL of the supernatant was added to 200 μL of Salkowski reagent (10 mM FeCl_3_, 97% reagent grade, and 34.3% perchloric acid, ACS grade) in duplicate. After incubating samples with the Salkowski reagent at room temperature for 30 min, the color change was recorded. A BioTek Synergy HT microplate reader was used to determine the absorbance (O.D.) at a single wavelength of 530 nm. To estimate the amount of indole related compounds at 530 nm, an IAA standard curve was generated by suspending IAA (Gibco Laboratories, Life Technologies, Inc., New York, USA) in 100% acetonitrile at a concentration of 1 mg/mL and diluting in LB medium or TSB to a concentration of 100, 50, 20, 10, 5, and 0 μg/mL. Sterile LB medium (with and without 5 mM L-tryptophan) and sterile TSB (with and without 5 mM L-tryptophan) were used as controls. The concentration of indole related compounds at 530 nm of the sterile control sample, either LB or TSB depending on the bacterial medium used, was subtracted from the concentration of indole related compounds at 530 nm of the bacterial samples to obtain a background subtracted concentration.

A full spectrum analysis from 440 to 600 nm, using a 1 nm interval, was performed to identify the wavelength of maximum absorbance. Full spectrum analysis was performed on bacterial samples grown in liquid LB medium supplemented with 5 mM L-tryptophan whereas free indole and free IAA (as references) were suspended in 100% acetonitrile. The wavelength of maximum absorbance (λ_max_) was calculated between 460 and 600 nm due to the high background signal observed at wavelengths shorter than 460 nm from addition of the Salkowski reagent to LB medium.

For determining the specificity of the Salkowski reagent, we tested IAA, indole-acetamide (IAM), indole-3-pyruvatic acid (IPA), ILA, indole-3-butyric acid (IBA), indole, indoxyl sulfate, tryptophol, and tryptophan. The compounds were suspended in 100% acetonitrile, HPLC grade, before diluting in LB medium, which did not contain 5 mM L-tryptophan due to the high absorbance background tryptophan generates when performing a spectrum analysis from 440 to 600 nm wavelength.

### Extraction of indole related compounds

For extraction of indole related compounds, bacterial strains from glycerol stocks were streaked onto an LB agar plate and grown at 28°C. A single colony was used to inoculate a starter culture of 6 mL liquid LB medium, supplemented with 5 mM L-tryptophan, and grown at 28°C and 240 rpm. After 24 h, the starter culture was used to make a 60 mL culture of liquid LB medium, supplemented with 5 mM L-tryptophan, at OD_600_ 0.01. The cultures were grown at 28°C and 240 rpm. After 24 h, the supernatant was collected by centrifugation at 10,000 × g for 5 min. For the spike sample, 300 μg of free IAA was added to 60 mL of LB medium supplemented with 5 mM L-tryptophan. All of the samples were then acidified with 1 N HCl to a pH of 2.7. The samples were then separated into 20 mL aliquots for biological triplicates. A Sep-Pak C18 cartridge (360 mg sorbent, 55–105 μm particle size) was prepared for each sample by washing with 10 mL of 100% acetonitrile followed by 10 mL of water. The acidified supernatant was passed through the C18 cartridge. The C18 cartridge was then washed with 10 mL of water and eluted with 5 mL of 80% (v/v) acetonitrile. The eluate was vacuum concentrated and then stored at−20°C until ready for LC/MS. Concentrated samples were dissolved in 1 mL of 100% acetonitrile. The samples were sonicated twice for 15 min and then centrifuged at 14,000 rpm for 5 min to remove any solid particles. A standard curve was generated by making IAA solutions of 0.5, 1, and 10 ng/μL in 100% acetonitrile. Acetonitrile of HPLC grade and HCl of ACS grade was used for the experiment and water was prepared from Millipore Synergy 185.

### Detection of IAA by LC/MS

Samples were separated and analyzed by a UPLC/MS system including the Dionex® UltiMate 3000 RSLC ultra-high pressure liquid chromatography system, consisting of a workstation with ThermoFisher Scientific's Xcalibur v. 4.0 software package combined with Dionex®'s SII LC control software, solvent rack/degasser SRD-3400, pulseless chromatography pump HPG-3400RS, autosampler WPS-3000RS, column compartment TCC-3000RS, and photodiode array detector DAD-3000RS. After the photodiode array detector the eluent flow was guided to a Q Exactive Plus Orbitrap high-resolution high-mass-accuracy mass spectrometer (MS). Mass detection was full MS scan with low energy collision induced dissociation (CID) from 100 to 1,000 m/z in either positive or negative ionization mode with electrospray (ESI) interface. Sheath gas flow rate was 30 arbitrary units, auxiliary gas flow rate was 7, and sweep gas flow rate was 1. The spray voltage was 3,500 volts (−3,500 for negative ESI) with a capillary temperature of 275°C. The mass resolution was 140,000 and the isolation window was 0.4 mDa. Substances were separated on a Phenomenex™ Kinetex C8 reverse phase column, size 100 × 2 mm, particle size 2.6 mm, pore size 100 Å. The mobile phase consisted of 2 components: Solvent A (0.5% ACS grade acetic acid in LCMS grade water, pH 3–3.5), and Solvent B (100% Acetonitrile, LCMS grade). The mobile phase flow was 0.20 ml/min, and a gradient mode was used for all analyses. The initial conditions of the gradient were 95% A and 5% B; for 30 min the proportion reaches 5% A and 95% B which was kept for the next 8 min, and during the following 4 min the ratio was brought to initial conditions. An 8 min equilibration interval was included between subsequent injections. The average pump pressure using these parameters was typically around 3,900 psi for the initial conditions.

Putative formulas of IAA metabolites and other indole related compounds were determined by performing isotope abundance analysis on the high-resolution mass spectral data with Xcalibur v. 4.0 software and reporting the best fitting empirical formula. Database searches were performed using reaxys.com (RELX Intellectual Properties SA) and SciFinder (American Chemical Society).

## Results

### Isolation of bacterial endophytes

Duckweed tissues that were selected from the RDSC or collected from various locations in New Jersey, USA are of the genera *Wolffia, Lemna, Spirodela*, and *Landoltia* (Supplementary Table 1). Forty-seven strains of bacteria were isolated from 16 strains of surface sterilized duckweed tissue. Of the bacterial isolates, 62% belong to the Proteobacteria phylum, 23% belong to the Firmicutes phylum, 11% belong to the Actinobacteria phylum, and 4% belong to the Bacteroidetes phylum (Supplementary Table 1).

### Specificity of the salkowski reagent

We tested the Salkowski reagent on various commercially available indole related compounds to determine the specificity of the assay (Table 1). IAA, IAM, and IPA, resulted in a color change to pink when these compounds were mixed with the Salkowski reagent. The wavelength which resulted in the maximum absorbance increase was 530 nm. IBA resulted in a color change to orange with a wavelength of maximum absorbance increase at around 450 nm. Indoxyl sulfate resulted in a purple color change with a wavelength of maximum absorbance increase at around 560 nm and indole resulted in a brown color change with a wavelength of maximum absorbance increase at around 490 nm. Tryptophan, ILA and tryptophol resulted in no observable color change.

**Table 1:** Specificity of the Salkowski reagent was determined by testing indole related compounds.

**Compound**	**Color change**	**Wavelength of maximum absorbance (nm)**
Tryptophan	No color change	N/a
ILA	No color change	N/a
Tryptophol	No color change	N/a
IAA	Pink	530
IAM	Pink	530
IPA	Pink	530
IBA	Orange	450
Indole	Brown	490
Indoxyl sulfate	Purple	560

*A color change indicates detection of one or more indole related compound(s). The wavelength recorded indicates the wavelength of maximum absorbance. ILA, Indole-3-lactic acid; IAA, Indole-3-acetic acid; IAM, Indole-acetamide; IPA, Indole-3-pyruvic acid; IBA, Indole-3-butyric acid. N/a indicates not applicable result*.

### Colorimetric detection of indole related compounds

Using the Salkowski reagent as a colorimetric assay, we determined that 78.7% (37 out of 47) of the isolated DABs are capable of producing indole related compounds (Supplementary Table 2). Of those DABs producing indole related compounds, 81.1% are pink-type as observed by a color change from yellow to pink at a maximum absorbance increase at around 530 nm and 18.9% are brown-type as observed by a color change from yellow to brown at a maximum absorbance increase at around 480 nm (Table 2 and Supplementary Table 2). As a positive control, we performed the Salkowski assay on *A. brasilense* strains Sp7 and Sp245, which are well-studied IAA producing PGPBs. When the Salkowski reagent was applied to the supernatant of Sp7 and Sp245, there was a color change to pink with an increase in absorbance that peaks at 530 nm. The concentration of indole related compounds in the sample was estimated using an IAA standard curve (Supplementary Table 3). The concentration of indole related compounds produced by pink-type bacteria, as compared to no color change bacteria, are listed in Table 2.

**Table 2:** Estimation for the amount of indole related compounds detected by the Salkowski reagent in bacterial culture supernatants grown with 5 mM L-tryptophan.

**A**	**Pink-type**		
**Bacteria genus**	**Strain**	**ng/**μ**L of indole related compound**	**Standard deviation**
	Sterile TSB	0	0.425
	Sterile LB	0	0.187
*Pseudomonas*	DAB D4EL2	3.485	0.973
*Pseudomonas*	DAB D2FKM1	3.666	1.918
*Pseudomonas*	DAB D2EKK1	4.376	0.125
*Paenibacillus*	DAB 26A	4.392	1.280
*Rhodanobacter*	DAB D4FL3	4.458	0.913
*Microbacterium*	DAB 19B	4.959	1.378
*Paenibacillus*	DAB 5M	5.036	1.929
*Microbacterium*	DAB 33B	5.182	2.873
*Azospirillum*	Sp7	5.396	3.024
*Pseudacidovorax*	DAB 35E	5.696	3.406
*Paenibacillus*	DAB 5A	6.967	1.586
*Janthinobacterium*	DAB D4EL3	7.484	3.200
*Rhizobium*	DAB 36D	7.533	2.690
*Azosprillum*	Sp245	7.764	1.333
*Herbaspirillum*	DAB 5O	8.424	3.206
*Microbacterium*	DAB 19A	8.564	2.154
*Pseudomonas*	DAB 36E	9.541	0.178
*Rhizobium*	DAB 35A	10.911	4.830
*Herbaspirillum*	DAB 5S	10.957	2.858
*Paenibacillus*	DAB 4T	11.196	2.501
*Paenibacillus*	DAB 4X	11.452	2.154
*Paenibacillus*	DAB 15A	11.719	0.818
*Pseudomonas*	DAB 38C	12.660	0.797
*Herbaspirillum*	DAB 5E	18.581	0.578
*Bosea*	DAB D4EK4	19.739	4.463
*Microbacterium*	DAB 1A	20.693	3.059
*Microbacterium*	DAB 1D	21.972	3.802
*Acidovorax*	DAB 35F	22.071	5.571
*Azospirillum*	DAB 38E	53.204	17.241
*Rhizobium*	DAB 20A	54.233	6.825
*Rhizobium*	DAB 33A	77.145	9.031
*Azospirillum*	DAB 37A	78.160	5.183
**B**	**No color change**		
***Genus***	**Strain**	**ng/**μ**L of indole related compound**	**Standard deviation**
*Bacillus*	DAB 3D	0.586	0.459
*Bacillus*	DAB 27A	0.878	0.216
*Staphylococcus*	DAB 1B	0.746	0.273
*Bacillus*	DAB 2C	2.660	1.119
*Bacillus*	DAB 36A	1.868	0.198
*Bacillus*	DAB 36C	1.076	0.618
*Pseudomonas*	DAB 38D	1.521	0.524
*Rhodanobacter*	DAB D4EH1	2.016	0.729
*Rhodanobacter*	DAB D4FH1	0.729	0.792
*Rhodanobacter*	DAB D4EL1	1.125	0.374

*Background subtracted concentrations were obtained by taking the average concentration as calculated from the standard curve and subtracting the concentration of either the sterile TSB or sterile LB sample, depending on the medium used to culture the bacteria. Means and standard deviation were calculated using 3 biological replicates. **(A)** Mean concentration (ng/μL) of indole related compound and standard deviation of Sp7, Sp245, and pink-type DABs. **(B)** Mean concentration (ng/μL) of indole related compound and standard deviation of DABs resulting in no color change*.

### Synthesis of indole related compounds without exogenous L-tryptophan

**Figure 1:**
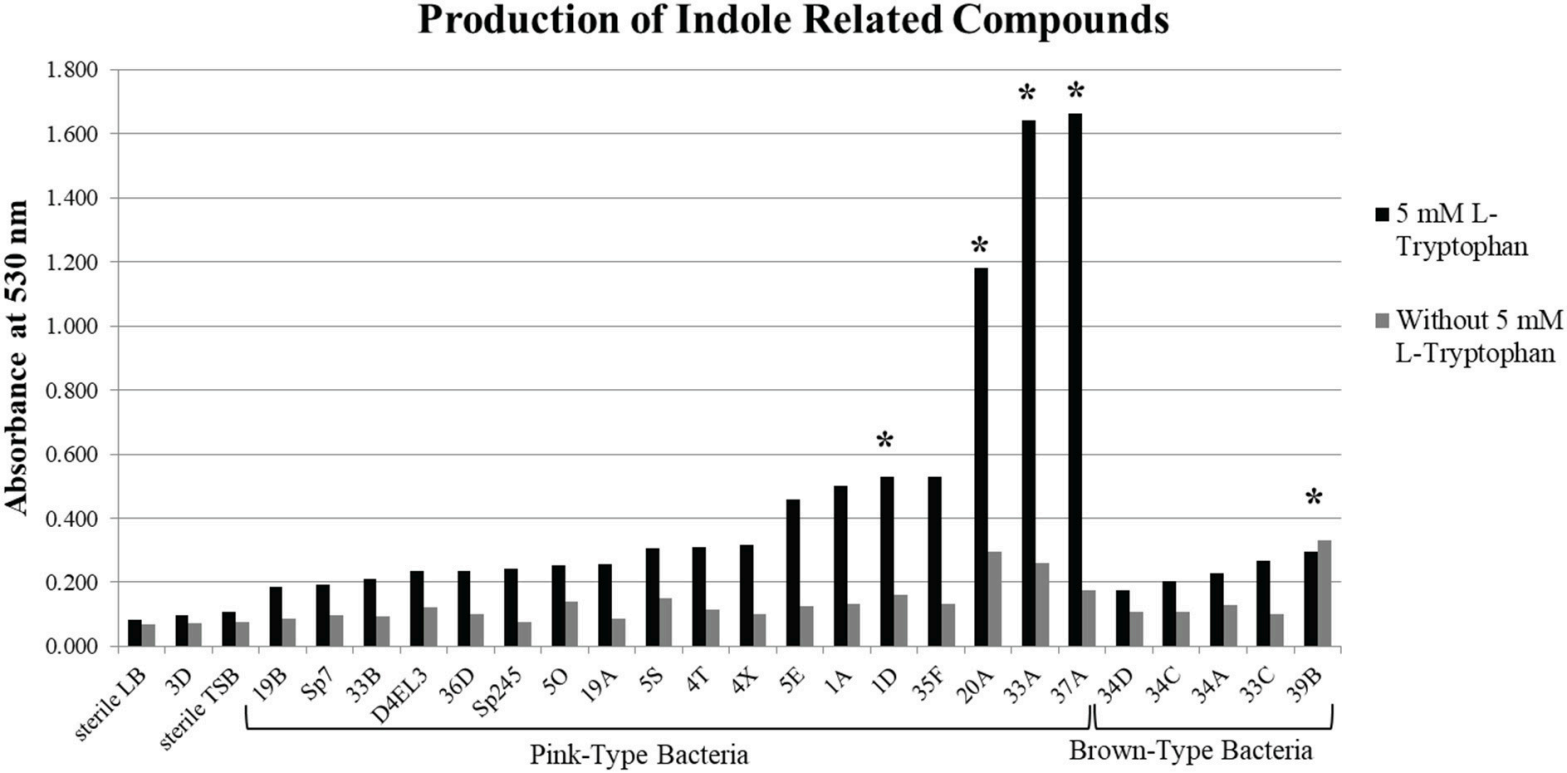
Production of indole related compounds from duckweed associated bacteria strains. Addition of Salkowski reagent to the bacterial supernatant of a subset of the pink-type and brown-type bacteria strains grown in medium with and without 5 mM L-tryptophan to measure the absorbance of indole related compounds at 530 nm. ^*^Indicates that the supernatant without 5 mM L-tryptophan resulted in an observable color change.

A subset of 18 pink-type and 5 brown-type bacterial strains were further screened for their ability to synthesize indole related compounds without 5 mM L-tryptophan supplemented into the growth medium. The full absorption spectrum from 400 to 600 nm of an IAA standard curve from the Salkowski assay reveals that a wavelength of maximum absorbance (λ_max_) cannot be detected at ≤ 5 μg/mL of IAA when no exogenous L-tryptophan is supplemented into the medium (Supplementary Figure 2). A λ_max_ cannot be detected at ≤ 10 μg/mL of IAA when 5 mM L-tryptophan is supplemented into the medium due to a high background absorbance. Four pink-type strains and one brown-type strain were able to synthesize indole related compounds without exogenous 5 mM L-tryptophan as determined by the color change after addition of the Salkowski reagent to the supernatant (Figure 1). This suggests that most of the DABs in our collection require an exogenous source of tryptophan to produce indole related compounds when cultured alone.

Using KEGG Mapper to analyze the complete genome sequences for 33 strains of our DAB collection, we identified tryptophan metabolism genes present in a subset of these bacterial genomes. Based on the estimated quantity of indole related compounds from the Salkowski assay, we compared the genomes of a pink-type top producer, a pink-type moderate producer and one that did not change color (DAB 37A, 1A, and 3D). We found that the pink-type top producer contained the most tryptophan metabolism-related genes of the three genomes compared. All three DABs contain a gene encoding a potential amidase enzyme, which may be able to convert IAM to IAA (Supplementary Figure 3 and Supplementary Table 4). Interestingly, the genome of DAB 3D which did not change color in the Salkowski assay, also appears to have this gene present, suggesting it may have the ability to convert IAM to IAA. However, based on the Salkowski assay, DAB 3D is not a producer of indole related compounds when grown alone in culture medium with or without addition of tryptophan.

### Potential correlation between production of indole related compounds and duckweed genus

Because of the large proportion of pink-type DABs and an unexpected proportion of DABs that turned brown, we wanted to determine whether there was an association between the type of indole related compound produced by the DABs and the duckweed genus from which these DABs were isolated. We calculated that of the 30 pink-type DABs, 19 or 63.3% are derived from *Lemna* species and of the 6 brown-type DABs that were isolated from genotyped duckweed strains, 4 or 66.7% are derived from *Wolffia* species (Supplementary Table 2). Pearson's chi square test of independence was used to calculate the null hypothesis that production of indole related compounds from DABs is independent of the duckweed genus that the DAB was isolated from. The obtained chi square value from the 3 by 4 contingency table is 20.3 with a degree of freedom of 6 and a *p*-value of 0.002 (Supplementary Table 5). The *p*-value is less than the critical value of 0.05 and therefore we reject our null hypothesis.

To determine which categories of the 3 by 4 contingency table are associated, two 2 by 2 contingency tables were created. The first 2 by 2 contingency table was used to test the null hypothesis that pink-type DABs are independent of the duckweed genus *Lemna*. The obtained chi square value is 4.30 with a degree of freedom of 1 and a *p*-value of 0.038 (Supplementary Table 5). The p value is less than the critical value of 0.05 and therefore we reject our null hypothesis. The second 2 by 2 contingency table was to test the null hypothesis that brown-type DABs are independent of the duckweed genus *Wolffia*. The obtained chi square value is 14.16 with a degree of freedom of 1 and a *p*-value of 0.0001 (Supplementary Table 5). The p value is much less than the critical value of 0.05 and therefore we reject our null hypothesis and conclude that there is a strong association between the brown-type DABs and the *Wolffia* genus, analogous to the association observed between the pink-type DABs and the *Lemna* genus. These results suggest that there is a significant correlation between the type of indole related compound produced by a particular DAB and the duckweed genus from which the DAB was isolated.

### Identification of indole related compounds by LC-MS

**Figure 2:**
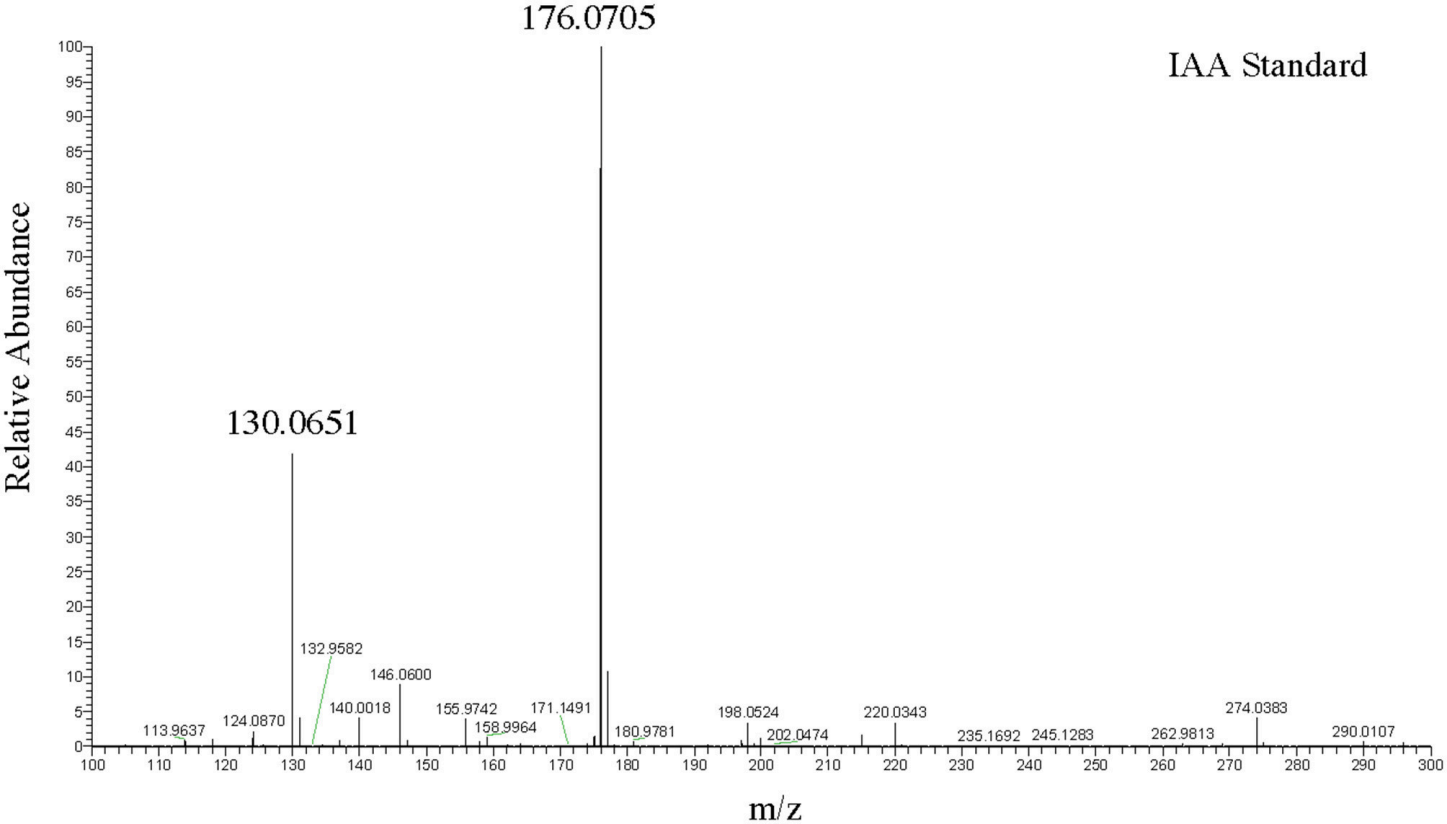
LC-MS positive ion scan spectrum of free IAA. One hundred nanogram/microliter of free IAA in 100% acetonitrile at a retention time of 9.94 min.

To more precisely validate the identity of the indole related compounds that are produced by the DABs, we first used LC-MS to determine whether the pink-type DABs were producing free IAA. The molecular weight of free IAA is 175 g/mol with positive ionization resulting in molecular ion at m/z 176 [M+H] and a fragment at m/z 130 (Figure 2). Negative ionization also resulted in molecular ion at m/z 174 [M–H] and a fragment at m/z 130. MS/MS was not performed due to the CID IAA fragmentation into an m/z 130 species representing the indole CH_2_ bridge component of IAA. Furthermore, fragmentation into an m/z 130 species from a larger compound at a different retention time from that of IAA led us to identify indole-lactic-acid (ILA) in one of the bacterial samples, indicating that the method could resolve specific indole related compounds.

**Figure 3:**
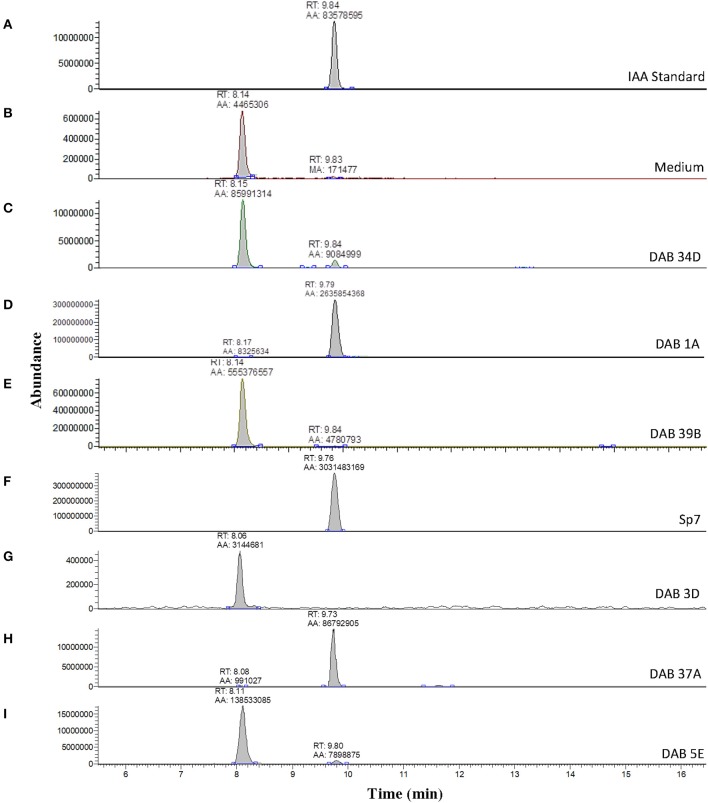
Validation of IAA production in DABs by mass spectrometry. LC-MS extracted positive ionization ion chromatograms at m/z 176.06 to m/z 176.08 ([M+H] of IAA). RT, retention time; AA, automatic area under peak; MA, manual area under peak. **(A)** Chromatogram (EIC) from an injection of 5 ng/μL of free IAA suspended in 100% acetonitrile showing a retention time of 9.84 min. **(B)** EIC showing a low background signal at the RT of free IAA in the LB medium sample. A second signal is detected at a retention time of 8.14 min in the LB medium sample. **(C)** EIC from the LB medium with brown-type *Vogesella* strain, DAB 34D. **(D)** EIC from the LB medium with pink-type *Microbacterium* strain, DAB 1A. **(E)** EIC from the LB medium with brown-type *Aeromonas* strain, DAB 39B. **(F)** EIC from the LB medium with pink-type *Azospirillum* strain, Sp7. **(G)** EIC from the LB medium with no color change *Bacillus* strain, DAB 3D. **(H)** EIC from the LB medium with pink-type *Azospirillum* strain, DAB 37A. **(I)** EIC from the LB medium with pink-type *Herbaspirillum* strain, DAB 5E. The scales on the Y-axis are automatically adjusted to show all the peaks in each of the chromatograms. Note the different scale on the chromatogram in **(B)** to show the lower quantity peaks.

A solution of 5 ng/μL of free IAA in 100% acetonitrile was used to determine the retention time of free IAA in our LC-MS system, which was approximately 9.84 min (Figure 3A) in extracted single ion chromatogram. The extracted ion chromatogram peak areas of m/z 176 are shown in Figures 3C–I for pink-type strains (Sp7, DAB 1A, DAB 37A, and DAB 5E), brown-type strains (DAB 34D and 39B), and no color change strain DAB 3D. The extracted ion chromatograms demonstrate that the brown-type strains, DAB 34D, and DAB 39B, produce small but detectable amounts of free IAA compared to the higher amounts observed in the pink-type strains. The background signal from the species with an m/z value of 176 in the medium with a shorter retention time of 8.0–8.1 min is not observed in the Sp7 and DAB 37A samples since the amount of IAA is much higher in these cases and the scale of the Y-axis is for a larger range. A free IAA standard was used to determine the HPLC UV absorbance signal at 280 nm for quantification (Supplementary Figure 4). The UV 280 nm absorbance signal for quantification of free IAA produced by Sp7, DAB 1A, DAB 37A, and DAB 3D are shown in Supplementary Table 6. Using a spike sample of 5 μg/mL free IAA in LB medium added just before our extraction and concentration protocol, we calculated a 61.5% recovery from the extraction process (Supplementary Table 6). Estimation of IAA production based on the Salkowski assay method suggested that our *Azospirillum* strain, DAB 37A, was a top IAA producer. However, quantification using HPLC, which has better resolution and sensitivity for IAA detection, suggests that *Microbacterium* strain, DAB 1A, and control *Azospirillum* strain, Sp7, produce higher levels of free IAA.

**Figure 4:**
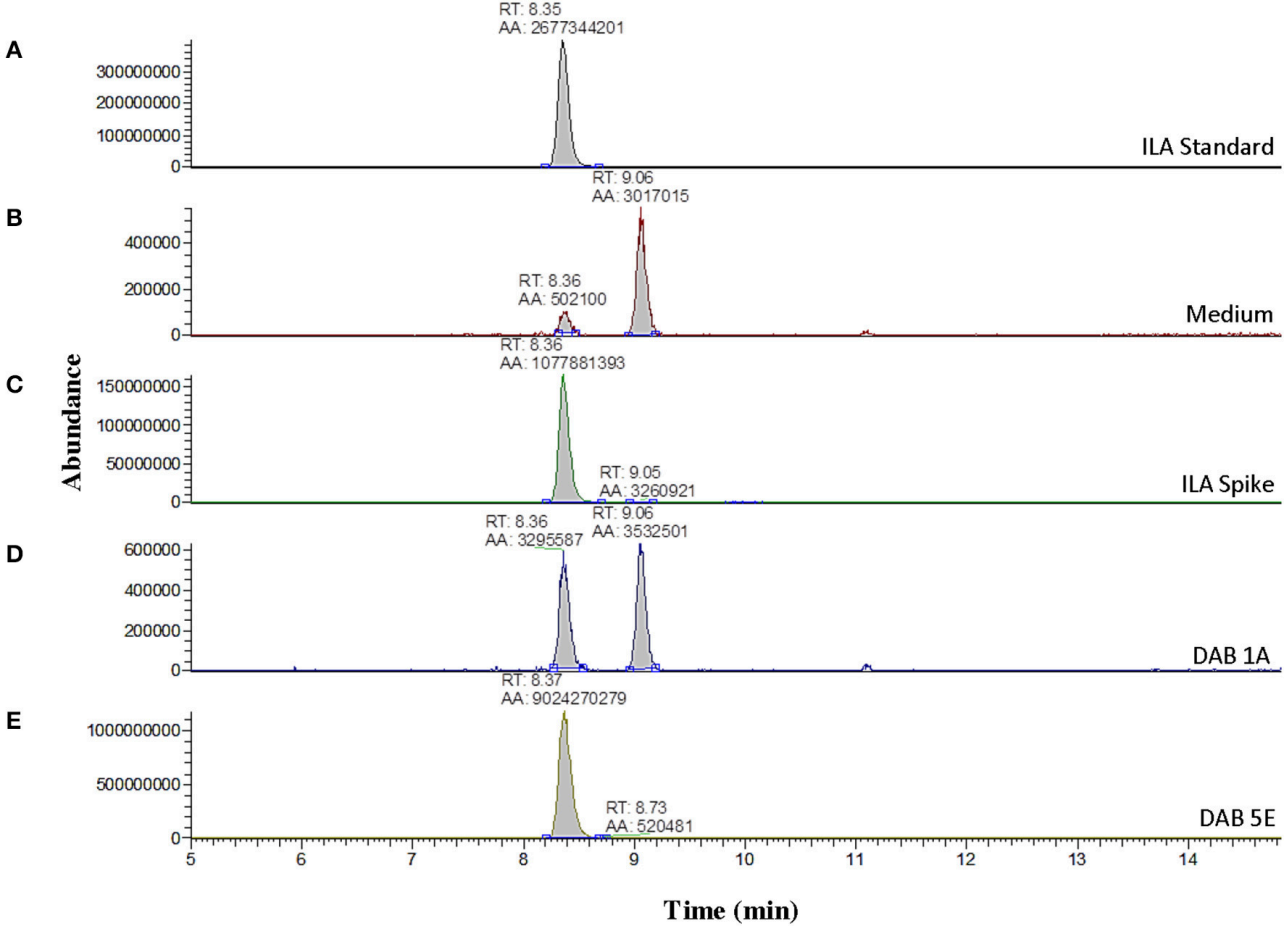
Detection of indole-3-lactic acid production in two DABs by mass spectrometry. LC-MS extracted negative ionization ion chromatograms at m/z 204.06 to m/z 204.07 ([M-H] of ILA). RT, retention time; AA, automatic area under peak. **(A)** EIC of an injection of 100 ng/μL of free ILA suspended in 100% acetonitrile showing a retention time of 8.35 min. **(B)** EIC showing a background signal at the RT of free ILA in the LB medium sample. A second signal is detected at a retention time of 9.06 min in the LB medium. **(C)** EIC from the LB medium spiked with 100 ng/μL of ILA. **(D)** EIC from the LB medium with pink-type *Microbacterium* strain, DAB 1A. **(E)** EIC from the LB medium with pink-type *Herbaspirillum* strain, DAB 5E. The scales on the Y-axis are automatically adjusted to show all the peaks in each of the chromatograms. Note the different scale on the chromatograms in **(B,D)** to show the lower quantity peaks.

Further LC-MS analysis revealed not only production of free IAA by the pink-type *Herbaspirillum* strain, DAB 5E, but also production of free ILA. This was detected by an m/z 130 fragment at a different retention time than IAA (Supplementary Figure 5). Free ILA did not result in a color change after addition of the Salkowski reagent (Table 1) and therefore, was only detectable using LC-MS. To verify the identity of the suspected ILA compound, a solution of 100 ng/μL of free ILA in 100% acetonitrile was used to determine the retention time of free ILA in our LC-MS system, which was approximately at 8.35 min (Figure 4A). Detection of free ILA with negative ionization resulted in a major peak at m/z 204 in extracted ion chromatogram. Extracted ion chromatograms at m/z 204 demonstrate that pink-type *Herbaspirillum* strain, DAB 5E produced free ILA in LB medium culture as well as a pink-type *Microbacterium* strain, DAB 1A, though at a much lower level (Figures 4D,E). These two DABs thus have an inverse ratio of IAA/ILA production with DAB 1A having a high IAA/ILA ratio while DAB 5E has a low IAA/ILA ratio (compare Figures 3, 4).

### Identification of the major salkowski-positive molecule in brown-type DABs as indole

When using KEGG Mapper to identify tryptophan metabolism genes present in the DAB bacterial genomes, we noted the presence of a gene encoding tryptophanase in all of the brown-type DABs (Supplementary Figure 3), excluding DAB 34D which does not have the complete genome sequenced yet. This enzyme produces indole from tryptophan and this product could react in the Salkowski assay to produce a brown color that would be consistent with our observations. We BLASTed the amino acid sequence of the *E. coli* tryptophanase provided by KEGG Mapper (K-12 MG1655) to the amino acid sequence database of our DAB genomes. The sequence was 84% identical to the predicted homolog in the brown-type *Aeromonas* strain, DAB 39B, with an E value of 0.0 (Supplementary Figure 6 and Supplementary Table 7). The other brown-type DABs with genome sequence available also contain a predicted coding sequence that is similar to the *E. coli* typtophanase, although the percent identity was lower (Supplementary Figure 6 and Supplementary Table 7).

**Figure 5:**
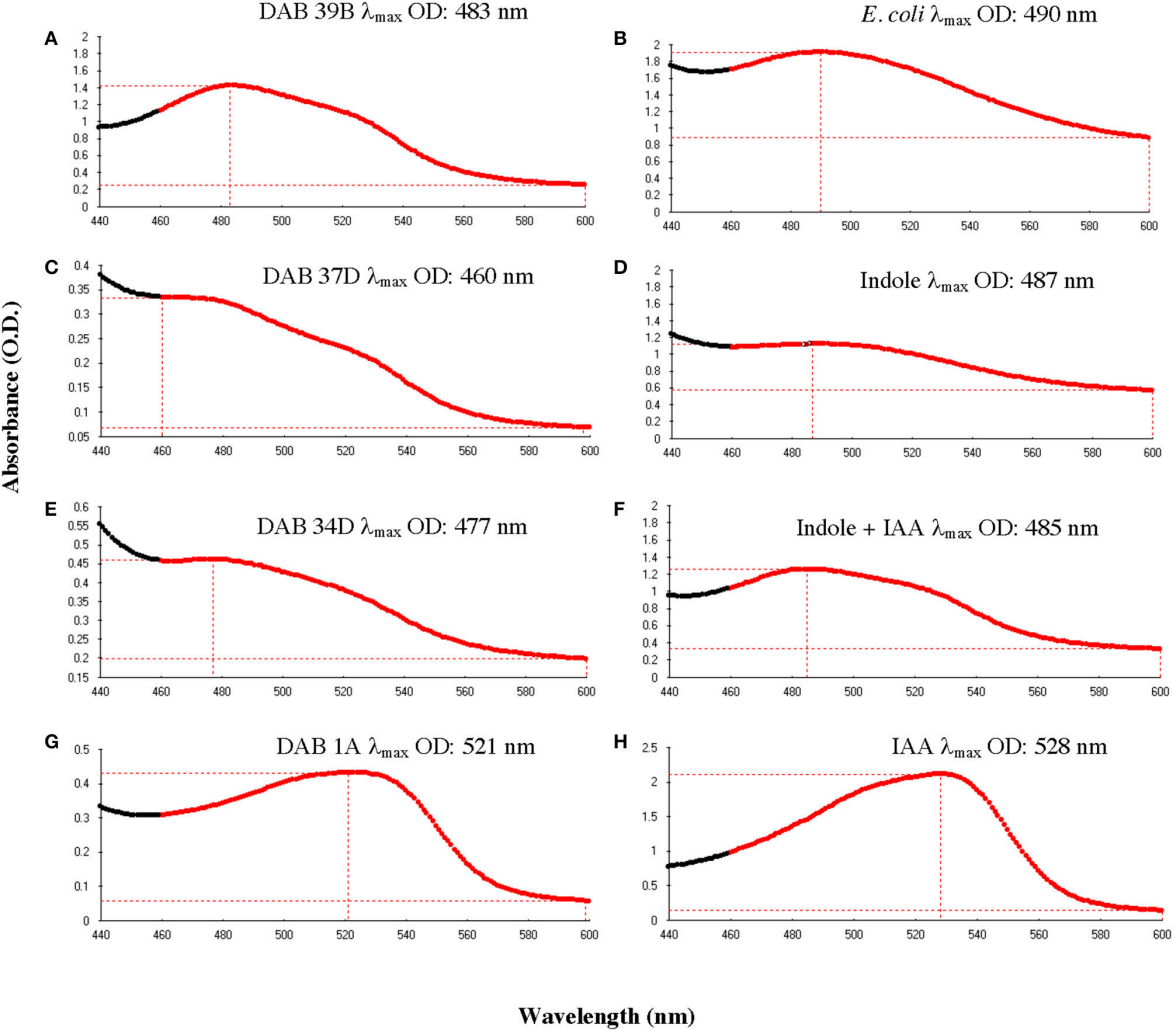
Comparison of absorbance spectra between DABs and other reference samples with the Salkowski assay. Absorbance spectrums from 440 to 600 nm to determine the wavelength at the maximum OD (λ_max_). **(A)** Brown-type *Aeromonas* strain, DAB 39B, has a λ_max_ at 483 nm and a minor peak at around 530 nm. **(B)** Known indole-producing *E. coli* strain, DH5 alpha, has a λ_max_ at 490 nm. **(C)** Brown-type *Chryseobacterium* strain, DAB 37D, has a λ_max_ at 460 nm and a minor peak at around 530 nm. **(D)** An LB solution containing 1 mg/mL of free indole has a λ_max_ at 487 nm. **(E)** Brown-type *Vogesella* strain, DAB 34D, has a λ_max_ at 477 nm and a potential minor peak at around 530 nm. **(F)** An LB solution containing a mixture of 1 mg/mL of free indole and 0.1 mg/mL of free IAA has a λ_max_ at 485 nm. **(G)** Pink-type *Microbacterium* strain, DAB 1A, has a λ_max_ at 521 nm. **(H)** An LB solution containing 0.1 mg/mL of free IAA has a λ_max_ at 528 nm.

To determine whether the Salkowski assay result of a brown color change is due to indole, we performed the Salkowski assay on *E. coli*, strain DH5-alpha, which is a well-studied indole-producing bacterium. The result was a color change from yellow to brown with a wavelength of maximum absorbance increase at 490 nm (Figure 5B). When the Salkowski reagent was added to LB medium containing 1 mg/mL of free indole compound, the color change was from yellow to brown with a wavelength of absorbance maximum at 487 nm (Figure 5D). The full spectra from reaction with supernatants from brown-type *Aeromonas* strain, DAB 39B, brown-type *Chryseobacterium* strain, DAB 37D, and brown-type *Vogesella* strain, DAB 34D, are shown in Figure 5 along with the full spectrum of LB medium containing both 1 mg/mL of free indole and 0.1 mg/mL of free IAA. There appear to be two peaks of absorbance corresponding to indole and IAA for DAB 39B, DAB 34D, and DAB 37D. Some of the DAB strains thus appear to be producing multiple indole related compounds during *in vitro* growth with potentially indole being one of them.

### Extraction and detection of indole from DAB strains by LC-MS

**Figure 6:**
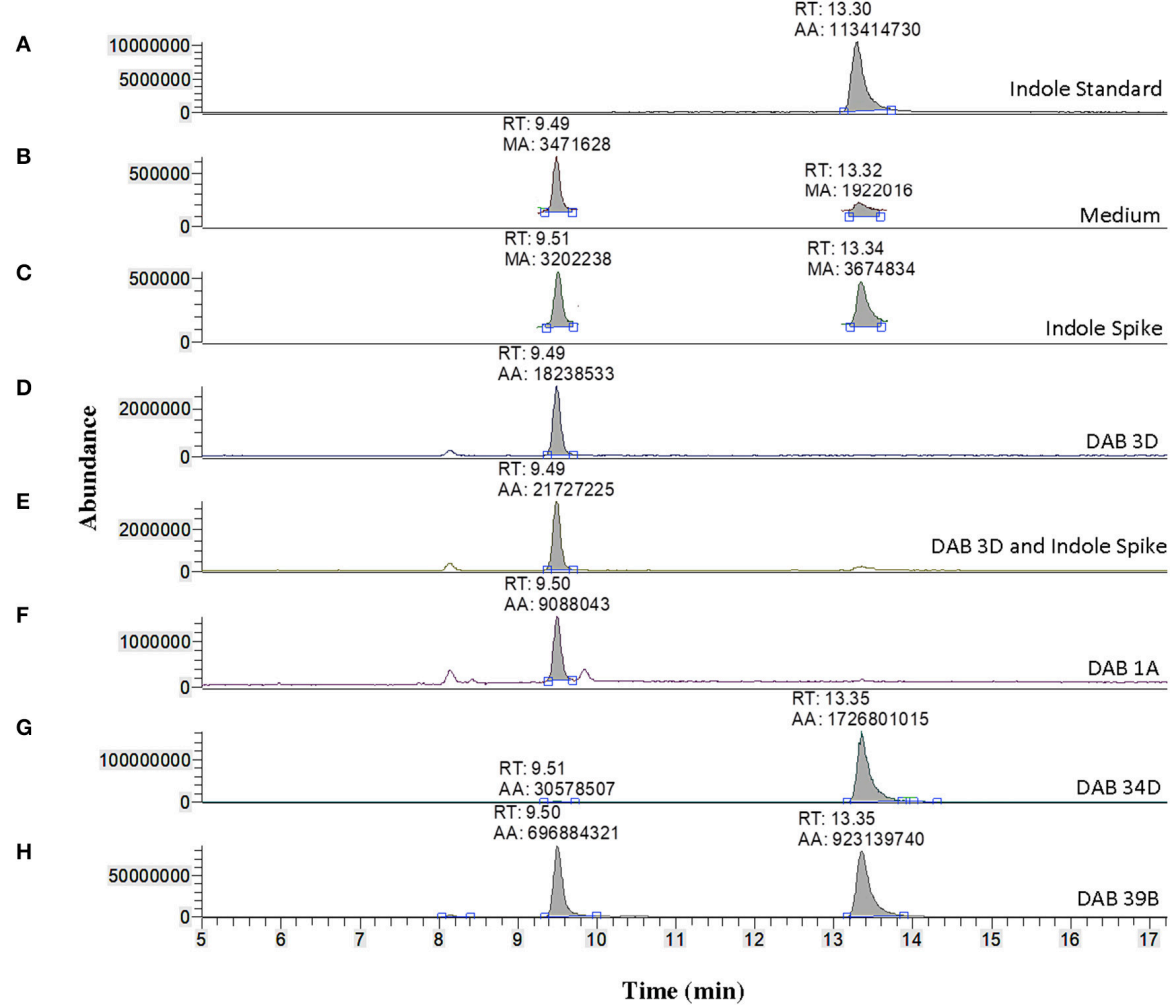
Identification of indole production in brown-type DABs by mass spectrometry. LC-MS extracted positive ionization ion chromatograms at m/z 118.06 to m/z 118.07 ([M+H] of indole). RT, retention time; AA, automatic area under peak; MA, manual area under peak. **(A)** EIC from an injection of 100 ng/μL free indole in 100% acetonitrile showing a retention time of 13.30 min. **(B)** EIC showing a low background signal at the RT of free indole in the LB medium sample. A second signal is detected at a retention time of 9.49 min in the LB medium. **(C)** EIC showing free indole in the LB medium spiked with 100 ng/μL of free indole. **(D)** EIC from the LB medium with non-color *Bacillus* strain, DAB 3D. **(E)** EIC from the LB medium with DAB 3D spiked with 100 ng/μL free indole. The indole peak area is < LoQ at the scale of the chromatogram. **(F)** EIC from the LB medium with pink-type *Microbacterium* strain DAB 1A, showing no detectable indole. **(G)** EIC from the LB medium with brown-type *Vogesella* strain DAB 34D, showing a clear indole signal. **(H)** EIC from the LB medium with brown-type *Aeromonas* strain DAB 39B, showing a clear indole signal. The scales on the Y-axis are automatically adjusted to show all the peaks in each of the chromatograms. Note the different scale on the chromatogram in **(B,C)** to show the lower quantity peaks.

We used LC-MS to more precisely validate the identity of the suspected indole compound produced in the brown-type DABs. A solution of 100 ng/μL of free indole in 100% acetonitrile was used to determine the retention time of free indole in our LC-MS system, which was approximately 13.30 min (Figure 6). Sample preparation and extraction of free indole were performed identically to the extraction of free IAA. Detection of free indole with positive ionization resulted in a major peak at m/z 118 in extracted ion chromatogram. Using the peak area at m/z 118 of the samples, we detected free indole present in the supernatants of the brown-type *Vogesella* strain, DAB 34D, and the brown-type *Aeromonas* strain, DAB 39B (Figure 6). No indole was detected with supernatant from the pink-type *Microbacterium* strain, DAB 1A. A peak corresponding to IAA was also detected in the supernatants of brown-type strains DAB 34D and DAB 39B, but at much lower levels compared to the pink-type strain, DAB 1A (Figure 3). Our results thus provided strong evidence for the identity of indole as the major compound produced by the brown-type DABs and not produced by the pink-type DABs.

## Discussion

The Salkowski colorimetric assay is traditionally used to detect the bacterial production of IAA. However, after testing various indole related compounds, IAM and IPA also resulted in a color change to pink with an absorbance maximum at 530 nm. Similar findings were previously reported in which IAA, IPA, and IAM all reacted with the Salkowski reagent to cause a pink color change, however sulfuric acid was used rather than hydrochloric acid (Glickmann and Dessaux, 1995). We used hydrochloric acid based on a previous report that it results in greater color intensity than sulfuric acid and thus could be more sensitive (Gordon and Weber, 1951). Our present study, along with a previous report, indicates that the Salkowski assay alone is not sufficient to distinguish which indole related compounds are produced by bacteria (Glickmann and Dessaux, 1995). This is also evident by the mass spec detection of ILA produced by pink-type *Herbaspirillum* strain, DAB 5E, which could not have been detected by the Salkowski reagent since it does not react with this particular indole related compound to produce a visible color change. Studies using the Salkowski assay should thus be cautious of these limitations and employ additional techniques such as mass spectrometry for specific detection and positive identification of the indole related compounds.

The isolated endophytic DABs were primarily from the phyla Proteobacteria, Firmicutes, and Actinobacteria, which has been similarly reported for the land plant, *Arabidopsis thaliana* (Bulgarelli et al., 2012; Lundberg et al., 2012). This indicates that the core microbiome of duckweeds may be quite similar to those of land plants such as the dicot *A. thaliana* and highlights the conservation of plant-microbe association mechanisms as far back as the divergence between monocots and dicots about 150 million years ago (Chaw et al., 2004). Of the 47 endophytic DABs used for the Salkowski assay in this study, 79% were capable of producing indole related compounds. This is similar to the suggested 80% of epiphytic bacteria reported in literature capable of producing IAA (Spaepen and Vanderleyden, 2011). However, the result in a brown color change after addition of the Salkowski reagent has not been widely reported. Mass spectrometry confirmed the presence of free IAA in the pink-type DABs and the presence of free indole in addition to free IAA in the brown-type DABs, although the levels of IAA in the latter cases are substantially lower. In addition, our genome analysis of non-Salkowski-reactive *Bacillus* strain, DAB 3D, revealed the presence of a potential amidase gene, which may convert IAM to IAA. However, the Salkowski assay and mass spectrometry did not detect the presence of free IAA in the DAB 3D supernatant. One explanation could be that the gene is not expressed when the bacteria is grown as a monoculture *in vitro*. Whether this is a pseudogene or if it is tightly regulated in DAB 3D to respond to specific cues remains to be determined. Future work is thus needed to determine whether some DABs are capable of producing free IAA when interacting with the duckweed hosts or in a community context.

In this study, we have also found that exogenous tryptophan is necessary for 79% of the tested DABs to produce indole related compounds during *in vitro* growth. The majority of the DABs may thus require exogenous tryptophan supplied by the plant to produce indole related compounds or they may have to be induced by the plant host to produce more tryptophan themselves. A previous study supports this notion that tryptophan-like compounds secreted by the plant root may stimulate IAA synthesis of PGPBs (Kamilova et al., 2006). Regardless of the cause for this requirement, our observation suggests that the DAB's phytohormone production capability during *in vitro* growth per se does not necessarily result in phenotypic effects of heightened auxin levels when inoculated onto the plant. Future experiments studying interactions of these DABs with various duckweed genera and environments will be necessary to better understand the dynamic control for this trait in different context of plant-microbe interaction. Our present results thus serve as a necessary foundation to better interpret future studies comparing the phenotypic output of different combinations of DAB and plant hosts. In this context, the availability of whole genome sequences for both DABs and high quality reference genomes for duckweed such as *Spirodela polyrhiza* (Michael et al., 2017) should enable the application of molecular tools to begin to characterize the signaling pathways involved.

The need to more closely examine mechanisms used by endophytic DABs to associate with the plant is further supported by the finding of an apparent correlation between the production of specific indole related compounds and the duckweed genus that the DAB was isolated from. Specifically, pink-type DABs are more likely to be associated with DABs isolated from *Lemna* species whereas brown-type DABs are overrepresented in DABs that were isolated from *Wolffia*. Enrichment of specific bacteria phyla by different plant host genotypes has been previously reported (Lundberg et al., 2012; Edwards et al., 2015; Haney et al., 2015). Our present work indicates one additional selection factor could be the particular type of genes for indole related compounds that the bacteria may be able to synthesize. The effect of IAA on plant root development has been well characterized and more recently studies have demonstrated that it could function as a signal molecule between bacteria in a community as well as between bacteria and the plant host (Spaepen and Vanderleyden, 2011). On the other hand, indole production by bacteria has been shown to be involved in a number of microbial processes including quorum sensing and biofilm formation (Lee and Lee, 2010). However its effect on plant health and development is poorly understood compared to that of IAA. *Wolffia* plants are morphologically distinct from *Lemna* species in that they are usually comprised of a more spherical frond and are rootless. Thus, architecturally, colonization of *Wolffia* plants by DABs may pose different challenges and different strategies/mechanisms, such as formation of a biofilm, could be needed in the bacteria. One possible explanation for our findings is that either the duckweed or the endophytic bacteria is selecting for a symbiont in which the appropriate resources it requires, whether it may be a specific indole related compound or a particular plant morphology, could be utilized and enable colonization of the microbe on the plant host. More studies will be necessary to uncover the role of indole producing DABs on the health of different duckweed genera and to delineate the function for the indole that could be produced by these bacteria.

Finally, we believe that understanding a potential coevolution between duckweeds and their associated endophytes is critical for selecting the appropriate combination of bacteria when designing synthetic bacterial communities. We may utilize bacteria to target specific or multiple duckweed species and therefore, it will be useful to understand how the different duckweed genera interact with various bacterial strains in order to distinguish general and species-specific rules and pathways. This will be important for researchers trying to improve large scale duckweed farming in open, non-sterile conditions for wastewater treatment, biofuel or bioplastic production, and animal feed supplement, which currently remains important challenges to overcome.

## Author contributions

EL conceived the experiments. EL, JX, KA, SL, and SG performed isolation and genotyping of bacterial strains and duckweed strains. SG performed the Salkowski test and genome analysis. SG prepared extraction of auxin related compounds for mass spectrometry. AP performed mass spectrometry. SG wrote the paper. KA, SL, AP, and EL provided input and revisions to the manuscript.

### Conflict of interest statement

The authors declare that the research was conducted in the absence of any commercial or financial relationships that could be construed as a potential conflict of interest.

## References

[B1] Aziz R. K. Bartels D. Best A. A. DeJongh M. Disz T. Edwards R. A.. (2008). The RAST server: rapid annotations using subsystems technology. BMC Genomics 9:75. 10.1186/1471-2164-9-75 18261238 PMC2265698

[B2] Baker G. C. Smith J. J. Cowan D. A. (2003). Review and re-analysis of domain-specific 16S primers. J. Microbiol. Methods 55, 541–555. 10.1016/j.mimet.2003.08.009 14607398

[B3] Bashan Y. de-Bashan L. E. (2010). How the plant growth-promoting bacterium *Azospirillum* promotes plant growth - a critical assessment. Adv. Agron. 108, 77–136. 10.1016/S0065-2113(10)08002-8

[B4] Bianco C. Imperlini E. Calogero R. Senatore B. Amoresano A. Carpentieri A.. (2006). Indole-3-acetic acid improves *Escherichia coli's* defences to stress. Arch. Microbiol. 185, 373–382. 10.1007/s00203-006-0103-y 16555073

[B5] Borisjuk N. Chu P. Gutierrez R. Zhang H. Acosta K. Friesen N. (2015). Assessment, validation and deployment strategy of a two-barcode protocol for facile genotyping of duckweed species. Plant Biol. 1, 42–49. 10.1111/plb.12229 25115915

[B6] Brettin T. Davis J. J. Disz T. Edwards R. A. Gerdes S. Olsen G. J.. (2015). RASTtk: a modular and extensible implementation of the RAST algorithm for building custom annotation pipelines and annotating batches of genomes. Sci. Rep. 5:8365. 10.1038/srep08365 25666585 PMC4322359

[B7] Bulgarelli D. Rott M. Schlaeppi K. Ver Loren van Themaat E. Ahmadinejad N. Assenza F.. (2012). Revealing structure and assembly cues for Arabidopsis root-inhabiting bacterial microbiota. Nature 488, 91–95. 10.1038/nature11336 22859207

[B8] Chakravorty S. Helb D. Burday M. Connell N. Alland D. (2007). A detailed analysis of 16S ribosomal RNA gene segments for the diagnosis of pathogenic bacteria. J. Microbiol. Methods 69, 330–339. 10.1016/j.mimet.2007.02.005 17391789 PMC2562909

[B9] Chaw S. M. Chang C. C. Chen H. L. Li W. H. (2004). Dating the monocot-dicot divergence and the origin of core eudicots using whole chloroplast genomes. J. Mol. Evol. 58, 424–441. 10.1007/s00239-003-2564-9 15114421

[B10] Cheng J. J. Stomp A.-M. (2009). Growing duckweed to recover nutrients from wastewaters and for production of fuel ethanol and animal feed. Clean Soil Air Water 37, 17–26. 10.1002/clen.200800210

[B11] Edwards J. Johnson C. Santos-Medellin C. Lurie E. Podishetty N. K. Bhatnagar S.. (2015). Structure, variation and assembly of the root-associated microbiomes of rice. Proc. Natl. Acad. Sci. U.S.A. 112, E911–E920. 10.1073/pnas.1414592112 25605935 PMC4345613

[B12] Glickmann E. Dessaux Y. (1995). A critical examination of the specificity of the Salkowski Reagent for indolic compounds produced by phytopathogenic bacteria. Appl. Environ. Microbiol. 61, 793–796. 16534942 10.1128/aem.61.2.793-796.1995 PMC1388360

[B13] Gordon S. A. Weber R. P. (1951). Colorimetric estimation of indoleacetic acid. Plant Physiol. 26, 192–195. 10.1104/pp.26.1.192 16654351 PMC437633

[B14] Haney C. H. Samuel B. S. Bush J. Ausubel F. M. (2015). Associations with rhizosphere bacteria can confer an adaptive advantage to plants. Nat. Plants. 1:15051. 10.1038/nplants.2015.51 27019743 PMC4806546

[B15] Kamilova F. Kravchenko L. V. Shaposhnikov A. I. Azarova T. Makarova N. Lugtenberg B. (2006). Organic acids, sugars, and L-tryptophane in exudates of vegetables growing on stonewool and their effects on activities of rhizosphere bacteria. Mol. Plant-*Microbe Interact*. 19, 250–256. 10.1094/MPMI-19-0250 16570655

[B16] Korner S. Vermaat J. E. (1998). The relative importance of *Lemna gibba* L., bacteria and algae for the nitrogen and phosphorus removal in duckweed-covered domestic wastewater. Water Res. 32, 3651–3661. 10.1016/S0043-1354(98)00166-3

[B17] Lee J. H. Lee J. (2010). Indole as an intercellular signal in microbial communities. FEMS Microbiol. Rev. 34, 426–444. 10.1111/j.1574-6976.2009.00204.x 20070374

[B18] Lui P. Nester E. W. (2006). Indoleacetic acid, a product of transferred DNA, inhibits *vir* gene expression and growth of *Agrobacterium tumefaciens* C58. Proc. Natl. Acad. Sci. U.S.A. 103, 4658–4662. 10.1073/pnas.0600366103 16537403 PMC1450227

[B19] Lundberg D. S. Lebeis S. L. Paredes S. H. Yourstone S. Gehring J. Malfatti S.. (2012). Defining the core *Arabidopsis thaliana* root microbiome. Nature 488, 86–90. 10.1038/nature11237 22859206 PMC4074413

[B20] Martino P. D. Fursy R. Bret L. Sundararaju B. Phillips R. S. (2003). Indole can act as an extracellular signal to regulate biofilm formation of *Escherichia coli* and other indole-producing bacteria. Can. J. Microbiol. 49, 443–449. 10.1139/w03-056 14569285

[B21] Michael T. P. Bryant D. Gutierrez R. Borisjuk N. Chu P. Zhang H.. (2017). Comprehensive definition of genome features in *Spirodela polyrhiza* by high-depth physical mapping and short-read DNA sequencing strategies. Plant J. 89, 617–635. 10.1111/tpj.13400 27754575

[B22] Overbeek R. Olson R. Pusch G. D. Olsen G. J. Davis J. J. Disz T.. (2013). The SEED and the rapid annotation of microbial genomes using subsystems technology (RAST) Nucleic Acids Res. 42, D206–D214. 10.1093/nar/gkt1226 24293654 PMC3965101

[B23] Santoyo G. Moreno-Hagelsieb G. Orozco-Mosqueda M. C. Glick B. R. (2016). Plant growth-promoting bacterial endophytes. Microbio. Res. 183, 92–99. 10.1016/j.micres.2015.11.008 26805622

[B24] Spaepen S. Vanderleyden J. (2011). Auxin and plant-microbe interactions. Cold Spring Harb. Perspect. Biol. 3:a001438. 10.1101/cshperspect.a001438 21084388 PMC3062209

[B25] Spaepen S. Vanderleyden J. Remans R. (2007). Indole-3-acetic acid in microbial and microorganism-plant signaling. FEMS Microbiol. Rev. 31, 425–448. 10.1111/j.1574-6976.2007.00072.x 17509086

[B26] Suzuki W. Sugawara M. Miwa K. Morikawa M. (2014). Plant growth-promoting bacterium *Acinetobacter calcoaceticus* P23 increases the chlorophyll content of the monocot *Lemna minor* (duckweed) and the dicot *Lactuca sativa* (lettuce). J. Biosci. Bioeng. 118, 41–44. 10.1016/j.jbiosc.2013.12.007 24468072

[B27] Yamaga F. Washio K. Morikawa M. (2010). Sustainable biodegradation of phenol by *Acinetobacter calcoaceticus* P23 isolated from the rhizosphere of duckweed *Lemna aoukikusa*. Environ. Sci. Technol. 44, 6470–6476. 10.1021/es1007017 20704249

