# Supplementary material for: Bacterial Production of Indole Related Compounds Reveals Their Role in Association Between Duckweeds and Endophytes
Source: Front Chem. 2018 Jul 12;6:265. doi: 10.3389/fchem.2018.00265 (PMC6052042; doi:10.3389/fchem.2018.00265)
Supplement: Supplementary file 2 [file Image_1.PDF]

Supplementary Material

Bacterial production of indole related compounds reveals their role in association between duckweeds and endophytes

Sarah Gilbert*, Jenny Xu, Kenneth Acosta, Alexander Poulev, Sarah Lebeis, and Eric Lam.

*** Correspondence:** Corresponding Author: Eric Lam; eric.lam@rutgers.edu

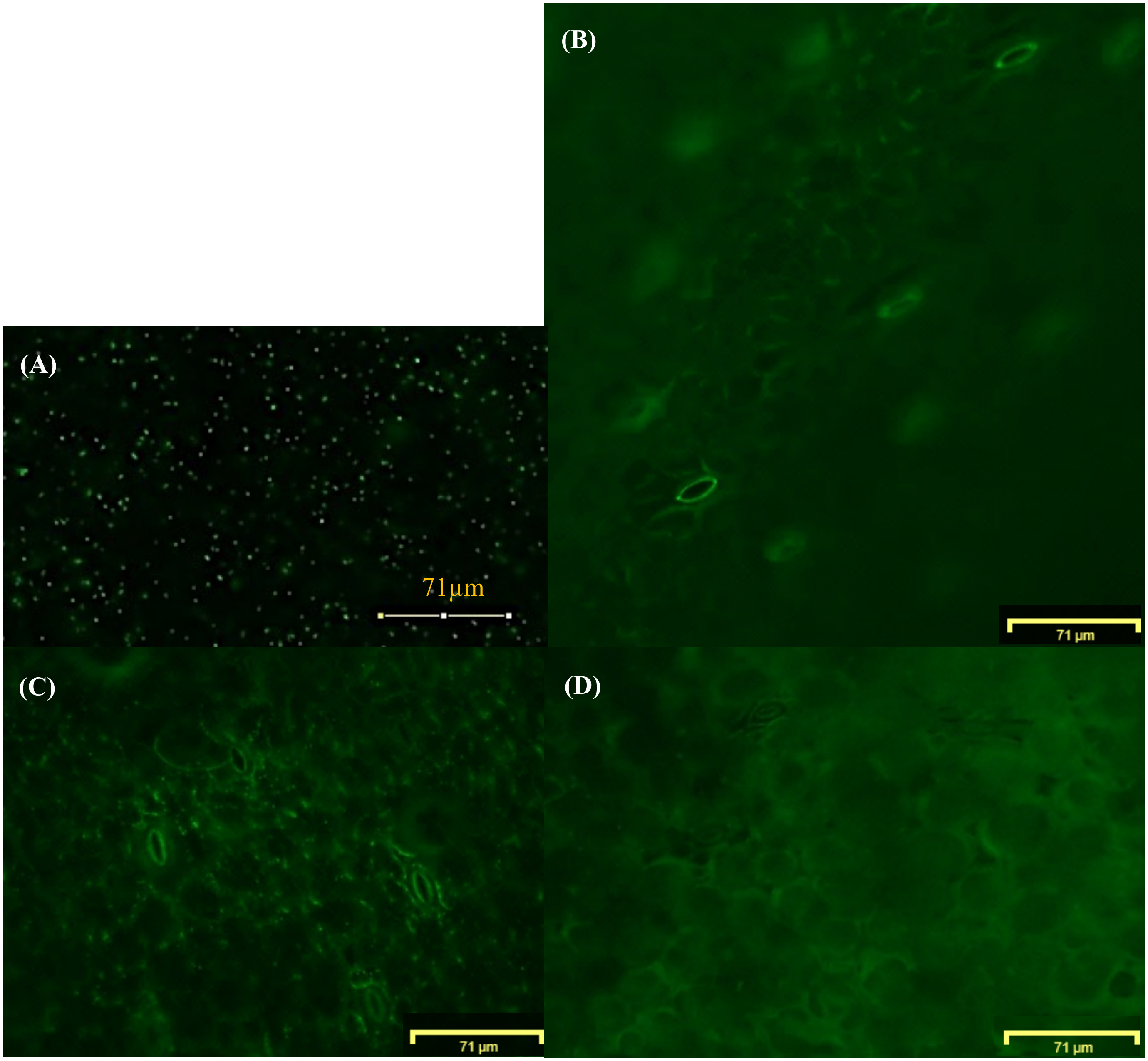

Supplementary Figure 1. Fluorescent microscopy images showing the method of surface sterilization to isolate endophytes from duckweed tissue. **(A)** DAB 1A for size reference. **(B)** Sterile *Lemna minor*, strain 370-DWC112. **(C)** *Lemna minor* strain 370-DWC112 inoculated with DAB 1A. **(D)** *Lemna minor* strain 370-DWC112 inoculated with DAB 1A and washed with sodium hypochlorite to remove surface bacteria.

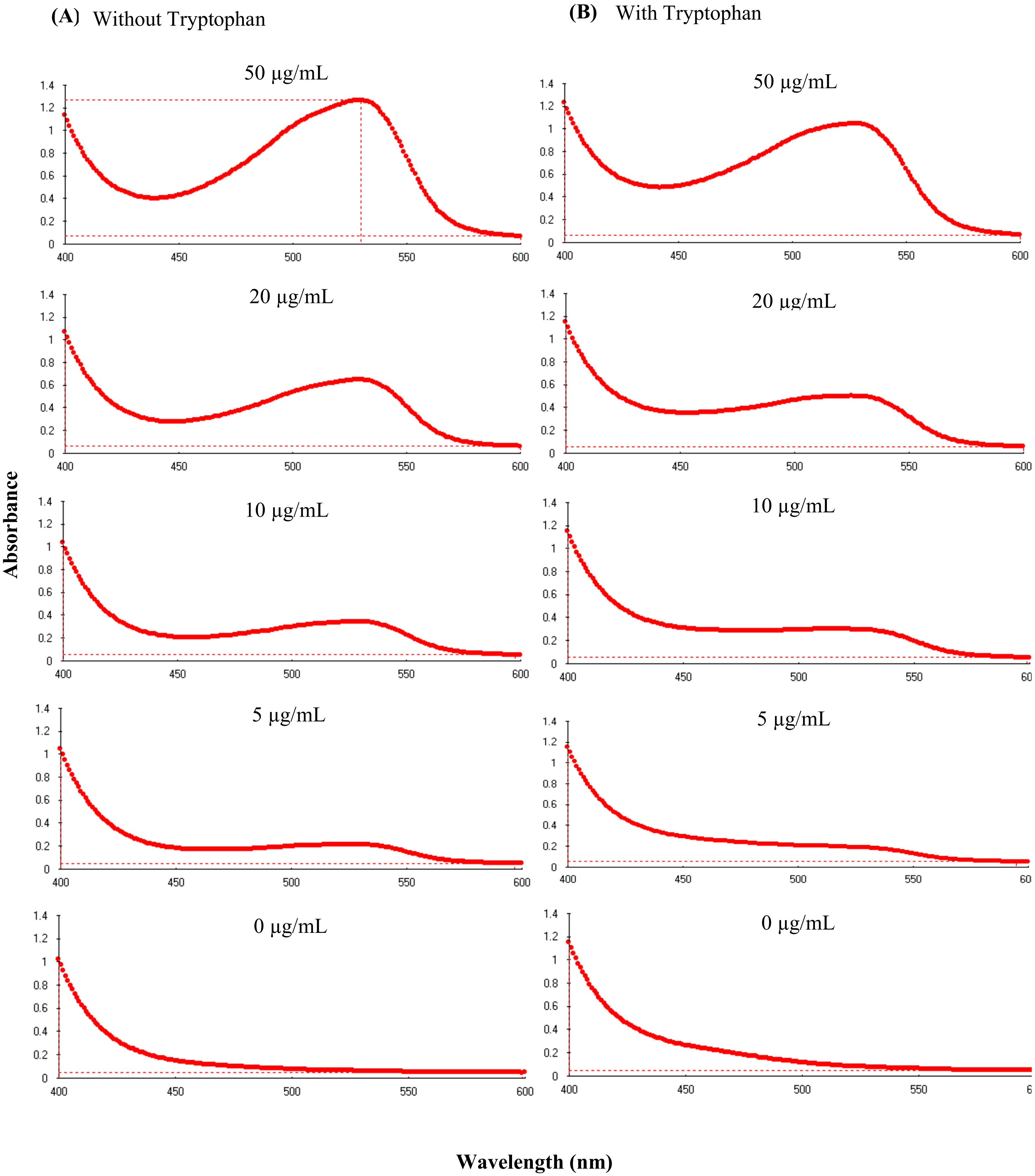

(A) Without Tryptophan
(B) With Tryptophan
50 µg/mL
20 µg/mL
10 µg/mL
5 µg/mL
0 µg/mL
Absorbance
Wavelength (nm)

Supplementary Figure 2. Absorbance spectra from 400 nm to 600 nm of IAA at 50 µg/mL, 20 µg/mL, 10 µg/mL and 5 µg/mL. **(A)** IAA standards were made in 100% acetonitrile and diluted into LB medium without exogenous L-tryptophan. **(B)** IAA standards were made in 100% acetonitrile and diluted into LB with 5 mM L-tryptophan. The absorbance spectra of the standard curves demonstrate that IAA has a maximum absorbance increase at 530 nm. Due to the high absorbance background generated by 5 mM L-tryptophan, a λ_{max} cannot be detected at ≤10 µg/mL of IAA when 5 mM of L-tryptophan is supplemented.

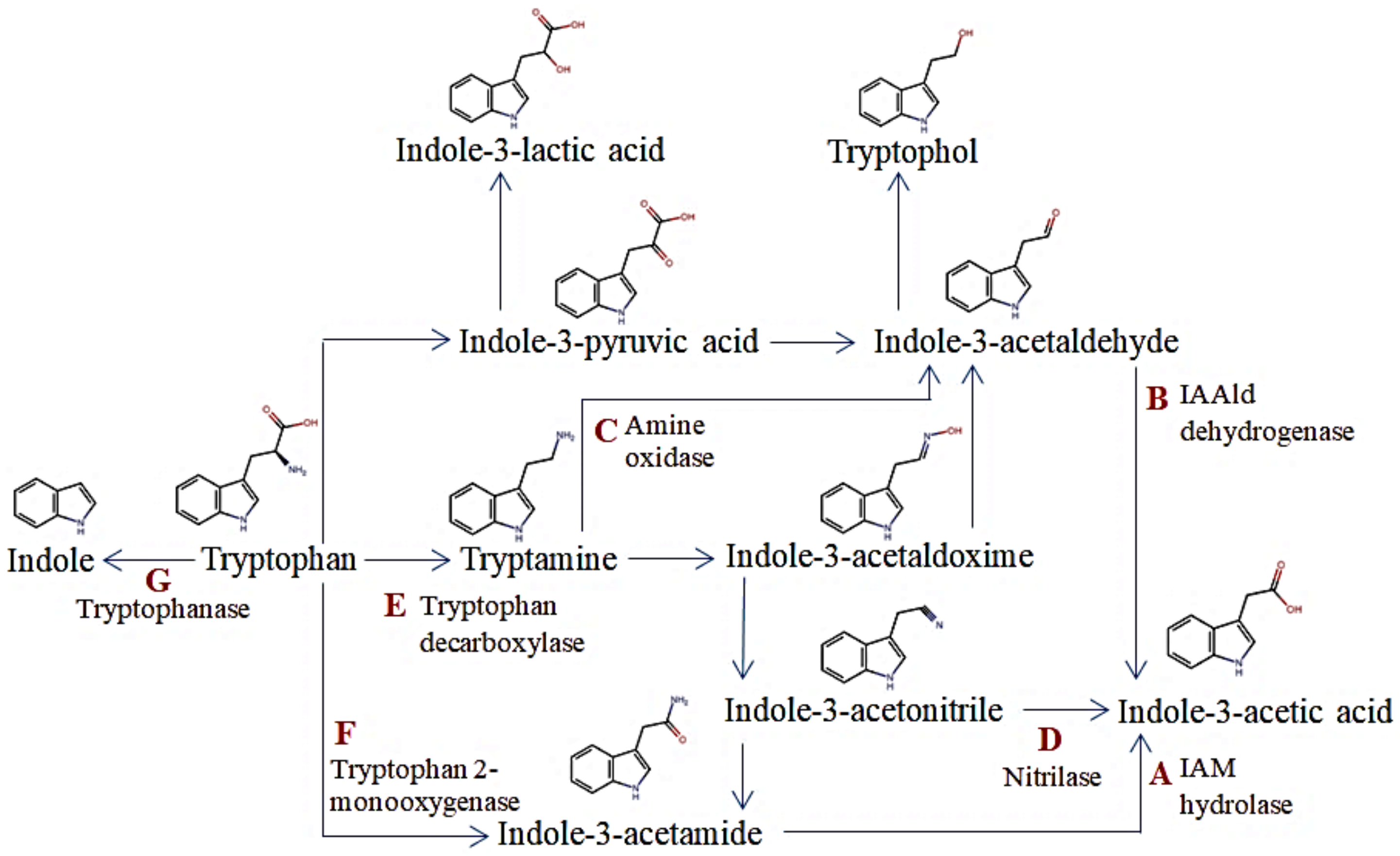

Supplementary Figure 3. Bacterial IAA biosynthesis and related enzymatic pathways. The enzymatic pathway diagram is modified from that of Spaepen and Vanderleyden, 2011. The enzymes involved in conversion of indole related compounds to IAA are labeled A-F. Enzyme G, tryptophanase, is involved in the conversion of tryptophan to indole.

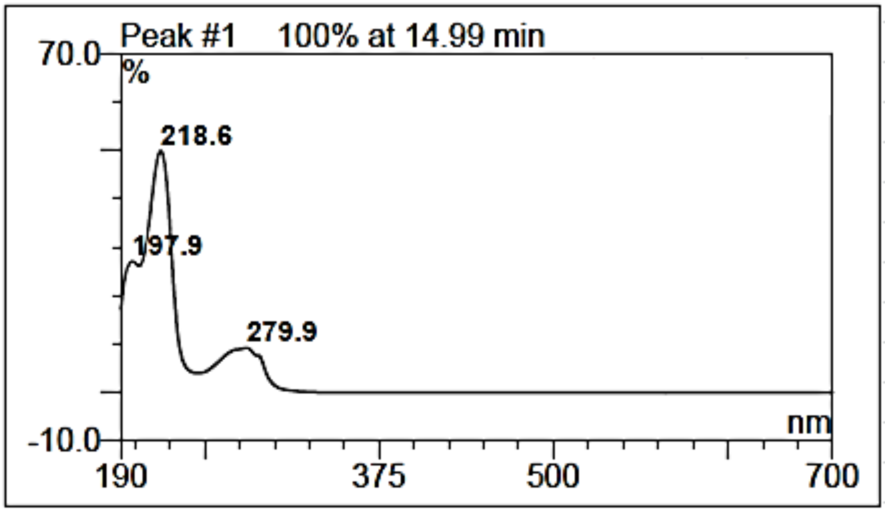

Supplementary Figure 4. Absorbance spectrum of 50 ng of free IAA in 100% acetonitrile using HPLC at a retention time of 14.99 minutes.

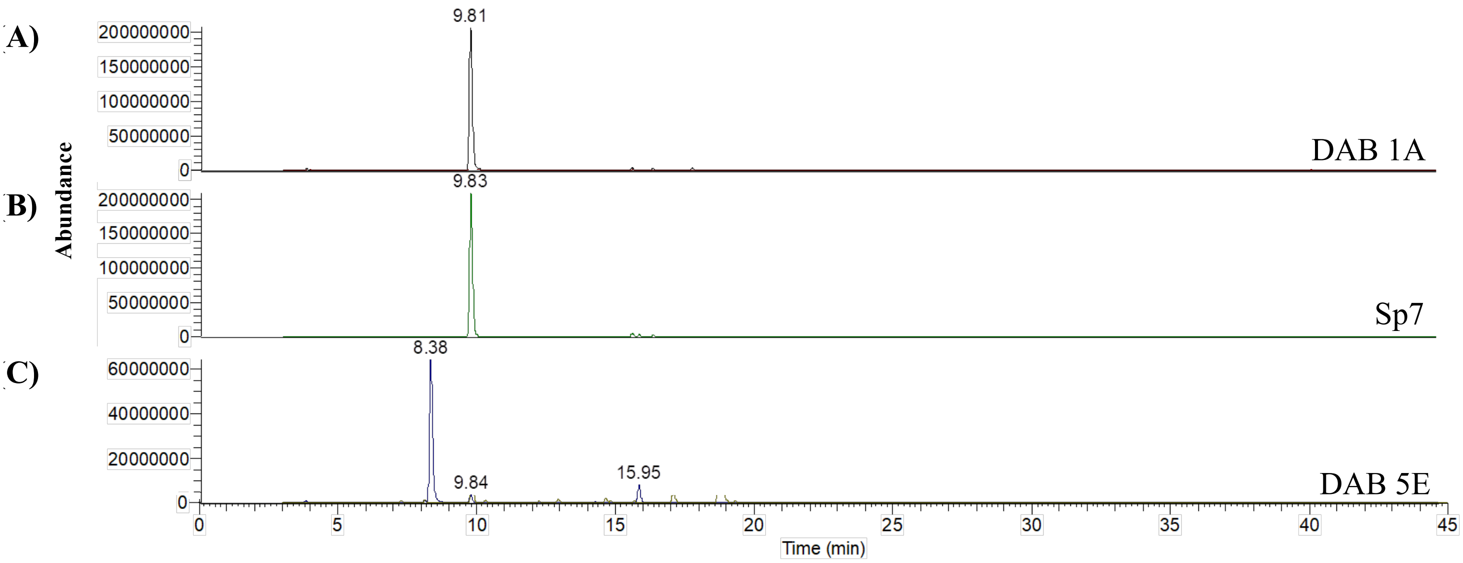

Supplementary Figure 5. LC-MS extracted positive ionization ion chromatograms at m/z 176.05 to m/z 176.07 and m/z 130.06 to m/z 130.08 ([M+H] of IAA). **(A)** Chromatogram (EIC) from the LB medium with DAB 1A. A signal is detected at a retention time of 9.81 minutes. **(B)** EIC from the LB medium with Sp7. A signal is detected at a retention time of 9.83 minutes. **(C)** EIC from the LB medium with DAB 5E. A signal is detected at a different retention time of 8.38 minutes.

(A)

```
DAB33C      --MNLPYAEPFRIKMVEEIYQSTREEREQWLKEANYNLFNLRSSHVYIDLLTDSGTGAMS      58
DAB37D      --MNLPYAEPFRIKMIEEIRQSTREEREQWLKEANYNLFNLKSSQVFIDLLTDSGTGAMS      58
DAB31B      ---MRRIPEPFRIKMVEPIKQTTGAERRAALEVAGWNPFLLLAEDVYIDLLTDSGTGAMS      57
DAB34A      ---MRRIPEPFRIKMVEPIKQTTGAERRAALEAAGWNPFLLLAEDVYIDLLTDSGTGAMS      57
DAB34C      ---MRRIPEPFRIKMVEPIKQTTGAERRAALEAAGWNPFLLLAEDVYIDLLTDSGTGAMS      57
E.coli      MENFKHLPEPFRIRVIEPVKRTTRAYREEAIIKSGMNPFLLDSEDVFIDLLTDSGTGAVT      60
DAB39B      MENFKHLPEPFRIRVIEPVKRTTREYRNTAILNAGMNPFLLDSEDVFIDLLTDSGTGAVT      60
                    *****:::* : ::*   *.  :  :. * * * :..*:***********::

DAB33C      DRQWSALMTGDESYAGSRSFEQLQKTVESITGFKYLLPTHQGRAAENVLFSVLVKEGD--      116
DAB37D      DRQWGALMMGDESYAGSRSFEQLHQTVKNITGFKYLLPTHQGRAAENVLFSVLVKEGD--      116
DAB31B      DRQWAGIMMGDEAYAGSRNFVELERTVRDIFGYQHVMPTHQGRGAEQILFPELVKRCKG-      116
DAB34A      DRQWAGIMMGDEAYAGSRNFVELERAVREIFGYQHVMPTHQGRGAEQILFPELVKRCKG-      116
DAB34C      DRQWAGIMMGDEAYAGSRNFVELERAVREIFGYQHVMPTHQGRGAEQILFPELVKRCKG-      116
E.coli      QSMQAAMMRGDEAYSGSRSYYALAESVKNIFGYQYTIPTHQGRGAEQIYIPVLIKKREQE      120
DAB39B      QDMQAAMMRGDEAYSGSRSFHALRNAVQDIFGYALTIPTHQGRGAEQIYIPVLIKKREKE      120
            :   ..:* ***:*:***.:  * .:*..* *:   :******.**:: :  *:*. .

DAB33C      --------VVPGNSHFDTTKGHIEIRKAHAIDCTIDEAFDINDLHPFKGNINLEKLEEVY      168
DAB37D      --------VIPGNSHFDTTKGHIEFRKAHAIDCTIDEAFDINDLHPFKGNINLEKLEEVY      168
DAB31B      -----KAPVFISNYHFDTTKAHVELAGARAINLLTPKALDTTAPYAWKGDFDLPRLTDTI      171
DAB34A      -----KAPVFISNYHFDTTKAHVELAGARAINLLTPKALDTTAPYAWKGDFDLPRLTDTI      171
DAB34C      -----KAPVFISNYHFDTTKAHVELAGARAINLLTPKALDTTAPYAWKGDFDLPRLTDTI      171
E.coli      KGLDRSKMVAFSNYFFDTTQGHSQINGCTVRNVYIKEAFDTGVRYDFKGNFDLEGLERGI      180
DAB39B      KGLDRKKMVALSNYFFDTTQGHTQINCCAARNVYTKEAFDTSVTADFKGNFDLAKLEAAI      180
                    *  .* .****:.* ::  . . :    :*:*      :**:::*  *

DAB33C      KSHPKENIPFCLITITCNSSGGQPVSLENMKAVKALSDQYGIPVFFDSARFAENAYFIKK      228
DAB37D      KSHPKENIPFCLITITCNSSGGQPVSLENMKAVKELSEKYGVSVFFDSARFAENAYFIKK      228
DAB31B      ETMGAANVAGIIITVTCNSAGGQPVSMANMEAVSELARRHHIPVVIDAARFAENAWFIKE      231
DAB34A      ETLGSDNVAGIIITVTCNSAGGQPVSMANMEAVSKLARRHHIPVVIDAARFAENAWFIKE      231
DAB34C      ETLGSDNVAGIIITVTCNSAGGQPVSMANMEAVSKLARRHHIPVVIDAARFAENAWFIKE      231
E.coli      EEVGPNNVPYIVATITSNSAGGQPVSLANLKAMYSIAKKYDIPVVMDSARFAENAYFIKQ      240
DAB39B      AEAGAANVPYIVSTITCNSAGGQPVSIANLKAVYEIARQHDIPVIMDSARFAENAYFIQQ      240
                  *:   : *:*.**:******: *::*:  :: :: : *.:*:*******:**::

DAB33C      REVGQENRSIKEICKEIFSYGDGMTMSSKKDGLVNIGGFIALNNE---EVFRKASNFTII      285
DAB37D      REAGQENKSIKEICKEIFSYGEGMTMSSKKDGLVNIGGFIALNNE---EVFRKASNFTII      285
DAB31B      RDPAYAKCSIKEIVRQMFALGDIFTMSAKKDGLVNIGGLCCFKED--LDLFRAVQVRCVA      289
DAB34A      RDPAYAKRSIKEIVRQIFALGDIFTMSAKKDGLVNIGGLCCFKED--LDLFRAVQVRCVA      289
DAB34C      RDPAYAKRSIKEIVRQIFALGDIFTMSAKKDGLVNIGGLCCFKED--LDLFRAVQVRCVA      289
E.coli      REAEYKDWTIEQITRETYKYADMLAMSAKKDAMVPMGGLLCMKDDSFFDVYTECRTLCVV      300
DAB39B      REPGYSNWTIEQITKEAYKYADGLAMSAKKDAMVPMGGLLCFKDESMLDVYNECRTLCVV      300
            *:    . :*::* :: :  .: ::**:***.:* :**: .::::   :::       :

DAB33C      YEGFITYGGMAGRDMAALAVGLDEATEFAYLESRISQVEYLGNKLIEYGIPVQKPIGGHA      345
DAB37D      YEGFITYGGMAGRDMAALAVGLDEATEFAYLESRISQVEYLGNKLIEYGIPVQKPIGGHA      345
DAB31B      MEGFVTYGGLAGRDMAALAIGLREGMDEEYLTYRISQVAYLGERLAEAGIPIQTPTGGHA      349
DAB34A      MEGFVTYGGLAGRDMAALAIGLREGMDEEYLTYRISQVAYLGERLAEAGIPIQTPTGGHA      349
DAB34C      MEGFVTYGGLAGRDMAALAIGLREGMDEEYLTYRISQVAYLGERLAEAGIPIQTPTGGHA      349
E.coli      QEGFPTYGGLEGGAMERLAVGLYDGMNLDWLAYRIAQVQYLVDGLEEIGVVCQ-QAGGHA      359
DAB39B      QEGFPTYGGLEGGAMERLAVGLYDGMRQDWLAYRIQQVQYLVDGLEAIGIVCQ-QAGGHA      359
             *** ****: *  *  **:** :.    :*  ** ** ** : *   *:  *   ****

DAB33C      VFIDSLNFLPNVSREEYPAQTLGLEIYKEAGIRTVEIGTLLADRDPATRENRYPKLELVR      405
DAB37D      VFIDSLNFLPKVAREEYPAQTLGLEIYKEAGIRTVEIGTLLADRDPETRENRYPKLELVR      405
DAB31B      VFVDAKKLLPHIPAEQFPAHALACELYLEGGVRGVEIGSLLLGRDPATGKQEAADFELLR      409
DAB34A      VFVDAKKLLPHIPAEQFPAHALACELYLEGGVRGVEIGSLLLGRDPATGKQEAADFELLR      409
DAB34C      VFVDAKKLLPHIPAEQFPAHALACELYLEGGVRGVEIGSLLLGRDPATGKQEAADFELLR      409
E.coli      AFVDAGKLLPHIPADQFPAQALACELYKVAGIRAVEIGSFLLGRDPKTGKQLPCPAELLR      419
DAB39B      AFVDAGKLLPHIPAGQFPAHALACELYKVAGIRAVEIGSLLQGRDPATGKQMACPAELLR      419
            .*:*: ::**::   ::**::*. *:*  .*:* ****::* .*** * ::      **:*

DAB33C      LAIPRRTYTNNHMDYIAAAIRNVYERREEIAKGYKITWEPEILRHFTVQLEKA-------      458
DAB37D      LAIPRRTYTNNHMDYIAAAIKNVYERREKIAKGYKITWEPELLRHFTVHLEKA-------      458
DAB31B      LTIPRRVYTRDHMDYVADCLIALKARASD-IRGLTFDYEPPLLRHFTARLKPV-------      461
DAB34A      LTIPRRVYTRDHMDYVADCLIAVKARASD-IRGLTFDYEPPLLRHFTARLKPV-------      461
DAB34C      LTIPRRVYTRDHMDYVADCLIAVKARASD-IRGLTFDYEPPLLRHFTARLKPV-------      461
E.coli      LTIPRATYTQTHMDFIIEAFKHVKENAAN-IKGLTFTYEPKVLRHFTAKLKEV-------      471
DAB39B      LTIPRATYTQTHMDFIIEAFQEVKKNAKN-IKGLEFTYEPPVLRHFTARFKEVETTAQGS      478
            *:***  .**. ***::   .:  :  .  .  :*  : :**  :*****.::: .
```

(B)

Genus	Strain	DAB 33C	DAB 37D	DAB 31B	DAB 34A	DAB 34C	K-12 MG1655	DAB 39B
Chryseobacterium	DAB 33C	100						
Chryseobacterium	DAB 37D	93.89	100					
Aeromonas	DAB 31B	50.66	50	100				
Aeromonas	DAB 34A	51.1	50	97.83	100			
Aeromonas	DAB 34C	51.1	50	97.83	100	100		
Escherichia coli	K-12 MG1655	44.96	45.61	54.13	54.35	54.35	100	
Aeromonas	DAB 39B	46.05	45.39	56.3	55.87	55.87	83.65	100

Supplementary Figure 6. Sequence comparison of the tryptophanase enzyme in brown-type DABs and *E. coli* genomes using Clustal Omega. **(A)** Amino acid sequence alignment. **(B)** Percent identity matrix.
